# Unveiling a Neurological Enigma: Cerebral Autosomal Dominant Arteriopathy With Subcortical Infarcts and Leukoencephalopathy (CADASIL) Presenting With Facial Palsy

**DOI:** 10.7759/cureus.60165

**Published:** 2024-05-12

**Authors:** Sakshi Jain, Vaishnavi Sirekulam, SudhaRani Kinthada, Rashi Bharat Patel, Nishthaben Naik, Shikha Jain, Tanzina Khan, Harmeet Gill, Neel Patel, Athmananda Nanjundappa, Lovekumar Vala, Chandu Siripuram

**Affiliations:** 1 Geriatrics, Hackensack University Medical Center, Hackensack, USA; 2 Medicine, Vijayanagar Institute of Medical Sciences, Ballari, IND; 3 Obstetrics and Gynecology, Rangaraya Medical College, Kakinada, IND; 4 Medicine, Tianjin Medical University, Tianjin, CHN; 5 Health and Family Welfare, Primary Health Center, Navsari, IND; 6 Medicine, MVJ Medical College and Research Hospital, Bengaluru, IND; 7 Internal Medicine, Bangladesh Medical College, Dhaka, BGD; 8 Medicine, HopeHealth, Florence, USA; 9 Internal Medicine, GMERS Medical College Gotri, Vadodara, IND; 10 Internal Medicine, MedStar Franklin Square Medical Center, Baltimore, USA; 11 Medicine, Shantabaa Medical College, Amreli, IND; 12 Hospital Medicine, Geisinger Medical Center, Scranton, USA

**Keywords:** infarct, hereditary, tia, stroke, notch3, cadasil

## Abstract

Cerebral autosomal dominant arteriopathy with subcortical infarcts and leukoencephalopathy (CADASIL) is an uncommon genetic disorder that affects small blood vessels in the brain. It leads to neurological symptoms, disability-adjusted life years, and difficult emotional and physical situations for patients and their families. As unusual brain symptoms appear, it becomes important to understand the different clinical manifestations of CADASIL. Our case report and review examine several cases to demonstrate different presentations and management strategies of CADASIL.

A 52-year-old male with a family history of strokes at a young age from his father and paternal grandfather presented to a neurology clinic for left facial droop and drooling. Brain magnetic resonance imaging showed extensive periventricular and subcortical white matter disease, including the external capsule and subcortical white matter of the temporal lobe. Findings were suggestive of small vessel vasculopathy. A cerebral angiogram showed that all large extra- and intracranial vessels were patent without evidence of aneurysm formation. There was no obvious evidence of beading of the distal intracranial vessels. Cerebrospinal fluid studies were normal. The NOTCH3 mutation was sent to test for CADASIL, which came back positive. The patient was started on aspirin (81 mg) and atorvastatin (20 mg) daily. The patient was counseled on the possibility of having an ischemic or hemorrhagic stroke. Aspirin and atorvastatin were continued, a neuropsychological evaluation was ordered, and CADASIL genetic counseling and testing were offered to him and his children. Over several years, patients developed several strokes and seizures due to infarcts. He also developed intraparenchymal hemorrhage complicated by dysphagia, requiring a feeding tube. Due to his severe physical debility, he was discharged to a nursing home for rehabilitation, where he did not improve with therapy and remained bedbound. He was discharged and sent home with his family.

CADASIL can present as a diagnostic challenge due to its common presentation with migraines, transient ischemic attacks, and strokes, with or without risk factors. This unique presentation of CADASIL with facial palsy highlights the importance of emerging atypical presentations and the need for a detailed history of neuroimaging, family history, and personal history of neurovascular events. By accurately diagnosing the condition, patients and families can be counseled on the disease course and genetics. Management requires a multidisciplinary approach with neurology, genetic counseling, physical therapy, psychology, and psychiatry if depression or anxiety is present, with the aim of improving the patient’s quality of life.

## Introduction

Cerebral autosomal dominant arteriopathy with subcortical infarcts and leukoencephalopathy (CADASIL) is a hereditary early-onset vascular disease causing recurrent ischemic subcortical infarcts. Common presentations include transient ischemic attacks and strokes (74.36%), cognitive impairment (69.23%), psychiatric disturbance (25.64%), and migraines (15.38%) [1]. In the realm of medical mysteries, CADASIL stands as a perplexing and devastating hereditary disease [2]. This case review aims to delve into the intricate details surrounding CADASIL, shedding light on its unique presentations, etiology, spectrum of clinical manifestations, diagnostic challenges, and potential therapeutic approaches [3].

Numerous previously published studies have explored the genetic variants associated with CADASIL and its diverse clinical presentations. Understanding the genetic landscape and the spectrum of clinical manifestations is crucial for accurate management and tailored approaches. The studies have identified specific mutations and polymorphisms linked to CADASIL, leading to the foundation of our knowledge about the genetic basis of this disorder. Additionally, a broad spectrum of clinical presentations highlights the complexity of CADASIL, making it essential for healthcare providers to consider a broad range of differentials when diagnosing and managing affected individuals.

This article has been presented as an abstract at the American Medical Association research challenge on October 18, 2023.

## Case presentation

**Figure 1:**
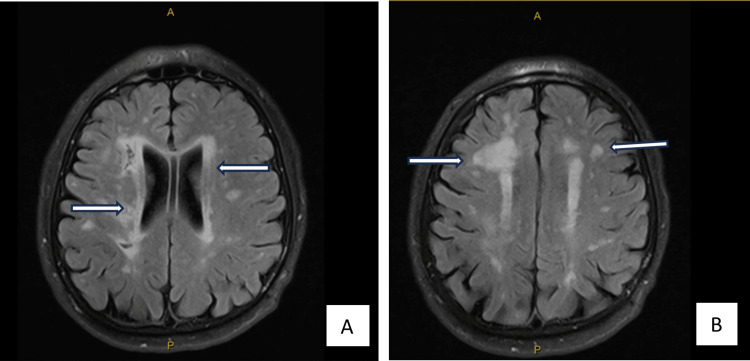
Brain MRI without contrast showing extensive periventricular and subcortical white matter disease, including external capsule (A; white arrows) and subcortical white matter of the temporal lobe (B; white arrows) MRI: magnetic resonance imaging

**Figure 2:**
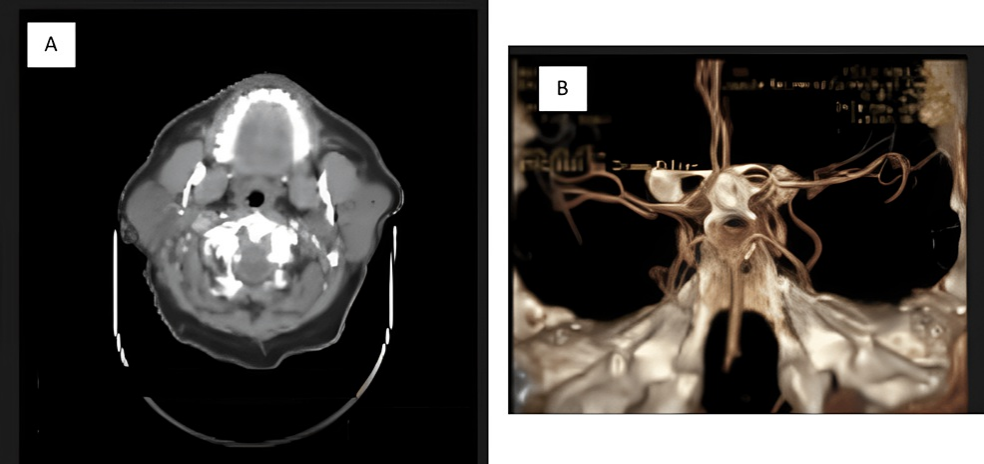
CT head and neck angiogram with and without intravenous contrast showing a normal brain with no abnormalities (A) and all large extra and intracranial vessels that are patent without evidence of aneurysm formation or beading of the distal intracranial vessels (B) CT: computed tomography

A 52-year-old male with a past medical history of type 2 diabetes presented to a neurology clinic with left facial droop and drooling for one week. He drank socially and did not have a history of smoking. His family history consisted of strokes at a young age in his father and paternal grandfather. Since facial droop was isolated to the lower face, a brain magnetic resonance imaging (MRI) was ordered, which showed extensive periventricular and subcortical white matter disease, including the external capsule and subcortical white matter of the temporal lobe (Figure 1) but did not show a stroke. MRI brain findings were suggestive of small vessel vasculopathy and warranted further investigation for an etiology. The differential diagnosis included demyelinating disease of the brain. The neurologist ordered a cerebral angiogram and lumbar puncture (LP) with cerebrospinal fluid (CSF) analysis for cell count, glucose, protein, cytology, oligoclonal bands, and IgG index. A computed tomography (CT) angiogram of the head and neck (Figure 2) showed that all large extra and intracranial vessels were patent without evidence of aneurysm formation. There was no obvious evidence of beading of the distal intracranial vessels. CSF studies were normal (Table 1). Due to these findings, small vessel ischemic disease was suspected, and a NOTCH3 mutation test was sent to test for CADASIL. The patient was started on 81 mg of aspirin and 20 mg of atorvastatin daily. The patient's result came back, and he was diagnosed with CADASIL with the NOTCH3 mutation showing a C>T transition at the nucleotide and codon positions at 499 and 141, respectively, resulting in the amino acid change arginine>cysteine. The patient was counseled on the possibility of having an ischemic or hemorrhagic stroke in the future. Aspirin and atorvastatin were continued, a neuropsychological evaluation was ordered, and CADASIL genetic counseling and testing were offered to him and his children.

**Table 1:** Patient's LP and serum electrophoresis results CSF: cerebrospinal fluid; LP: lumbar puncture

CSF study	Reference range	Patient’s results
Cell count		0
Clarity	Clear	Clear
Color	Colorless	Colorless
Volume		5.0 mL
RBC	<1 µL	76
WBC	0-8 µL	0
Character	Colorless	
Xanthochromia	None	
Glucose	45-75 mg/dL	62
Protein	15-45 mg/dL	36
Albumin	0-35 mg/dL	
Cytology	Neg	
Gram stain		No white blood cells; no organisms seen
CSF culture		No growth
Oligoclonal bands		No bands
Angiotensin-converting enzyme, CSF	<15 U/L	14 U/L
Venereal disease research laboratory, CSF		Nonreactive
IgG	694-1,618 mg/dL	1,180 mg/dL
Serum protein electrophoresis		
Protein	6.2-8.0 gm/dL	7.4 gm/dL
Albumin	3.7-4.8 gm/dL	4.6 gm/dL
Alpha 1	0.1-0.2 gm/dL	0.2 gm/dL
Alpha 2	0.6-1.0 gm/dL	0.7 gm/dL
Beta	0.6-1.1 gm/dL	0.9 gm/dL
Interpretation		The serum electrophoresis pattern is within normal limits

The patient was admitted to the hospital the following year with right-sided clonic movements and generalized seizures. He was determined to have localized seizures due to a previous cerebral infarction secondary to CADASIL. He was prescribed levetiracetam 750 mg twice a day and asked not to drive for six months. Later that year, he presented to the neurology clinic with dizziness. A CT head without contrast was performed, and he was told he had two new cerebral infarctions and benign paroxysmal positional vertigo. Clopidogrel 75 mg daily was added, and atorvastatin was increased to 80 mg daily with the continuation of levetiracetam 750 mg twice a day.

Three years after diagnosis, this fully functional patient, who was employed full-time, filed for disability. He was also diagnosed with essential hypertension and asked to have good blood pressure and diabetes control. Lifestyle modifications were encouraged for stroke prevention. The patient lost 50 pounds with diet and exercise. Nine years after diagnosis and follow-ups with multiple brain MRIs, he was started on L-arginine 2 g daily for one week, which was increased to 2 g twice daily on the second week and then 2 g three times daily.

Thirteen years after the initial diagnosis, the patient was admitted to the hospital after he fell at home with right-sided weakness. In the emergency department, his CT head showed left parietal intraparenchymal hemorrhage (IPH) with extension into the precentral gyrus and mild edema. CT head and neck angiogram were ordered, which was normal. The patient was admitted to the neurology intensive care unit for monitoring. He had a worsening of his examination with the National Institutes of Health Stroke Scale from 8 to 19. Repeat CT head showed IPH enlargement with no surgical intervention recommended. The hospital course was complicated by a newly diagnosed atrial fibrillation. Further workup included an MRI of the brain without contrast, redemonstrating left parietal IPH and two bilateral age-indeterminate ischemic infarcts. His echocardiogram showed an ejection fraction of 65% with no left atrial enlargement. Hospitalization was complicated by dysphagia secondary to IPH, which led to a feeding tube placement. He was stabilized and discharged to a nursing home for rehabilitation, where he did not progress with therapy and remained bedbound. He was discharged home with his family, who provided 24-hour care.

## Discussion

This case presents a highly unique clinical scenario because of the presenting feature of facial palsy. The patient is a 52-year-old male exhibiting symptoms of left facial droop and drooling. This presentation is noteworthy due to its atypical manifestation, as the patient's MRI brain was negative for a stroke. This unique presentation of facial palsy in CADASIL can be misleading initially, as it may resemble Bell’s palsy, a more common condition [4]. This similarity can lead to representative bias, where the initial assumption favors the more prevalent condition, namely acute stroke, which the initial brain MRI did not show. The patient's family history is particularly remarkable, as his father and paternal grandfather also had a history of strokes. This familial pattern strongly suggests an autosomal dominant inheritance pattern, which aligns with the genetic nature of CADASIL. In addition to the previously mentioned aspects, the brain MRI revealed extensive periventricular and subcortical white matter disease, particularly affecting the external capsule and subcortical white matter of the temporal lobe. These specific patterns of white matter involvement, along with the absence of aneurysm formation and beading of distal intracranial vessels on the cerebral angiogram and a positive NOTCH3 gene, strongly support the diagnosis of CADASIL.

There is ample evidence in the literature that suggests the association of NOTCH3 mutations [5]. The NOTCH3 gene provides instructions for producing a protein involved in the structure and function of blood vessels in the brain. Mutations involving the gene can lead to the accumulation of abnormal protein deposits called granular osmiophilic material. These form within the walls of small blood vessels, causing them to become thickened and narrowed. This limits blood flow and impairs oxygenation of brain tissue, leading to the characteristic clinical presentation and imaging findings.

Even with an atypical presentation of facial palsy, CADASIL was diagnosed accurately in this patient with a comprehensive history and evaluation, and appropriate treatment strategies were initiated to improve the patient’s quality of life despite various comorbid conditions (diabetes, atrial fibrillation), adding burden to the patient’s care. CADASIL cases can present a diagnostic challenge due to the diverse range of initial and ongoing presenting symptoms that can occur over time and affect different areas of the body. Therefore, a holistic approach involving specialists in neurology, cardiology, and medical genetics is necessary for accurate diagnosis and management. Table 2 describes various published cases on CADASIL, their findings, and management.

**Table 2:** Details of various published cases on CADASIL MRI: magnetic resonance imaging; LP: lumbar puncture; CT: computed tomography; DWI: diffusion-weighted imaging; LDL: low-density lipoprotein; HDL: high-density lipoprotein; NA: not applicable

Study	Patient details (age/sex)	Initial presentation	Diagnostic tests performed	Findings	Management/outcomes
Tojima et al., 2016 [6]	A 47-year-old male	The patient presented with cortical motor aphasia and right hemiplegia after fainting in the bathroom	MRI, carotid ultrasonography, transesophageal echocardiography, immunohistochemical examination of skin biopsy, electron microscopy, and NOTCH3 gene analysis	MRI revealed multiple high-intensity lesions in the bilateral hemispheres on diffusion-weighted imaging with low apparent diffusion coefficient values. No embolic source was detected by carotid ultrasonography or transesophageal echocardiography. Skin biopsy showed accumulation of the NOTCH3 extracellular domain in the small vessels. NOTCH3 gene analysis identified a novel Cys323Trp mutation in exon 6	Aphasia and hemiplegia disappeared within one month of stroke onset
Hsiao et al., 2015 [7]	A 69-year-old male	The patient developed confusion, drowsiness, right hemiparesis, and slurred speech following orthopedic surgeries	MRI, brain magnetic resonance angiography, echocardiography, 24-hour electrocardiography, and NOTCH3 gene analysis	Brain MRI: diffuse leukoencephalopathy on fluid-attenuated inversion recovery imaging and multiple acute lacunar infarcts over bilateral centrum semiovale and corona radiata presenting as hyperintense DWI lesions coinciding with reduced apparent diffusion coefficient signals. Brain magnetic resonance angiography demonstrated that all the major intracranial vessels were patent. Echocardiography and 24-hour electrocardiography were unremarkable. NOTCH3 gene analysis found a heterozygous missense mutation, c.1630C>T (p. Arg544Cys), in exon 11	Symptoms improved with supportive management including fluid hydration and antiplatelet therapy. The patient's mental status recovered in three days
Dunphy et al., 2019 [8]	A 35-year-old male	The patient presented with slurred speech and increased confusion after a fall leading to head trauma after consuming alcohol	ECG; biochemistry including hematological, renal, and clotting tests; CT head; MRI; LP and optic fundoscopy; and echocardiogram	ECG revealed a left ventricular strain pattern. Biochemistry, including hematological, renal, and clotting tests, was normal. CT head demonstrated cerebral atrophy. Multiple small old infarcts were noted at the head of the caudate nucleus with moderate background microvascular disease. MRI head demonstrated widened perivascular spaces with small lacunar infarcts. Extensive white matter abnormalities and microhemorrhages were present. LP and optic fundoscopy were normal. Echocardiogram revealed severe left ventricular hypertrophy	Treatment included blood pressure and ischemic stroke management with medications. The patient was referred for genetic counseling
Algahtan et al., 2020 [9]	A 40-year-old male	The patient presented with a two-year history of progressive cognitive decline, including impaired attention, memory, executive functions, and processing speed. He also had gait instability, urinary urgency, and recurrent attacks of migraine with aura	Biochemical and hematological screens, neurological examination, MRI, NOTCH3 mutations in skin biopsy, and electron microscopy were performed. Furthermore, genetic testing was performed on the patient (proband II-1) using whole exome sequencing after acquiring informed consent	Biochemical and hematological screens were unremarkable. Positive for dysdiadochokinesia, bilateral babinski’s sign, spastic legs, and incoordination in the limbs. MRI of the brain showed multiple scattered subcortical and periventricular white matter T2 and fluid-attenuated inversion recovery bright signal intensity lesions, which are symmetrically distributed. Within the white matter, the frontal and temporal lobes were the sites with the highest lesion load. Novel mutations in NOTCH3 were discovered. Workup for other possible causes for the patient's presentation, including central nervous system vasculitis, connective tissue diseases, cardiac emboli, and drug and substance abuse, was all unremarkable	NA
Spearman et al., 2017 [10]	A 59-year-old female	The patient presented with altered mental status, right-sided weakness, and dysarthria. She was found unresponsive on her couch	MRI, genetic testing, and lipid profile	MRI: faintly hyperintense foci on DWI, possibly indicating a subacute lacunar infarct in the left basal ganglia, right centrum semiovale, and left anterior and central pons. A stable chronic lacunar infarct was noted in the left genu of the corpus callosum. NOTCH3 gene testing was positive for heterozygous missense p.Arg141Cys and p.Pro68Leu mutations. Cholesterol: 154; HDL: 88; LDL: 45; and triglycerides: 106	She was placed on dual antiplatelet therapy. Her symptoms resolved during hospitalization, and she was discharged home
Da Silva et al., 2012 [11]	A 52-year-old male	The patient presented with a history of transient ischemic attacks, migraines with visual auras, cognitive dysfunction, paresthesias, and faciobrachial crural hemiparesis to the left side	Neurophysiological assessment, MRI, and genetic analysis	MRI: extensive areas of hypersignal in subcortical white matter, predominantly frontal, temporal, and parietal, in addition to compromised external and internal capsules, brain stem, and presenting lacunar infarcts in the temporal and right parietal regions. Morphometric analysis: percentage of frontal lobe lesions (41.8%). Genetic testing: direct DNA sequencing of exons 3 and 4 of the NOTCH3 gene (chromosome 19), which revealed a nucleotide substitution of arginine (CGC) to cysteine (TGC) at position 153 in exon 4 (c.535 C>T: R153C)	The patient was placed on medications to control blood pressure and diabetes
Ganesan et al., 2020 [12]	A 46-year-old male	The patient presented with a nine-month history of progressive right lower limb weakness and a three-month history of progressive unclear speech	MRI, CBC, random blood glucose, HbA1c, LDL, 24-hour Holter monitor skin biopsy, and molecular genetic test	MRI: extensive white matter disease within the subcortical regions including the anterior temporal pole and multiple lacunar infarcts. Gradient echo MRI images showed microhemorrhages. CBC and HbA1c were normal. Skin biopsy: electron-dense deposits of extracellular osmiophilic granular material adjacent to smooth muscle cells. Molecular genetic testing: heterozygous mutation in exon 6 of the NOTCH3 gene (NM_000435.2:c.994C>T), leading to a replacement of arginine by a cysteine residue at position 332 p.(Arg332Cys)	Treatment included aspirin and atorvastatin. He became more dysarthric with repeat brain MRI showing the progression of the white matter disease and a new right subcortical infarct. Aspirin was switched to clopidogrel
Lahkim et al., 2021 [13]	A 51-year-old male	The patient presented with a four-year history of recurrent migraine attacks, most often accompanied by scintillating scotoma-type aura and cheiro-oral paresthesias extending to the upper limb	Brain MRI, LP, cardiovascular workup, and genetic testing	Brain MRI: confluent hyperintense lesions in the periventricular and subcortical white matter related to leukoaraiosis and sequelae of ischemic lacunar lesions in the supra and infratentorial white matter. LP showed no abnormalities. Cardiovascular workup was normal. Genetic testing: positive for mutation in NOTCH3 gene	Treatment included antiplatelet agents and symptomatic treatment of migraines with level 2 analgesics and antiepileptics. His migraine frequency and severity of attacks decreased
Ashraf et al., 2022 [14]	A 33-year-old female	The patient presented with complaints of right upper and lower extremity paresthesia and a brief episode of aphasia	CT scan of the head, chest X-ray, specific anticoagulation tests, Doppler of carotid arteries, and brain MRI	CT head: mild bilateral white matter hypoattenuation and temporal lobe encephalomalacia. Brain MRI: acute disseminated encephalomyelitis and confluent relatively symmetric white matter signal disease process involving periventricular and prominent subcortical white matter tracts, but no associated abnormal diffusion signal or abnormal postcontrast enhancement	Treatment included medical management, and she was discharged on aspirin and atorvastatin with neurology follow-up

The series of diverse cases reported in Table 2 are all connected by NOTCH3 gene mutations, showcasing various neurological symptoms and patient demographics. In five of these cases, Tojima et al. [6], Hsiao et al. [7], Dunphy et al. [8], Spearman et al. [10], and Ganesan et al. [12], patients presented with slurred speech and right-sided hemiplegia with lacunar infarcts found on MRI brain. In three cases, Algahtan et al. [9], da Silva et al. [11], and Lahkim et al. [13], patients presented with migraines and an MRI brain negative for acute stroke. One case report by Ashraf et al. [14] describes a 33-year-old female with right upper and lower extremity paresthesia and a brief aphasic episode with a CT head and MRI brain negative for stroke. These case reports collectively reveal diverse symptoms resulting from NOTCH3 gene mutations, leading to various neurological manifestations such as motor and speech impairments, cognitive decline, and migraines. MRI scans consistently showed white matter abnormalities and, in some cases, infarcts. Treatment approaches were primarily symptomatic, lacking specific targeted therapies. Responses to treatment varied among patients, emphasizing the need for further research to develop more effective and tailored interventions for these conditions.

## Conclusions

CADASIL is a rare genetic condition that can present with various neurological symptoms that can resemble more common conditions such as stroke or Bell's palsy. It can also present atypically with facial palsy. It is important to perform a comprehensive medical and family history and have a high suspicion for CADASIL with a positive family history. By precisely diagnosing the condition, the patient and their families can be counseled regarding the progression of the disease and genetic predisposition.
